# The Preoperative Microbial Detection is No Prerequisite for the Indication of Septic Revision in Cases of Suspected Periprosthetic Joint Infection

**DOI:** 10.1155/2018/1729605

**Published:** 2018-06-21

**Authors:** Daniel Karczewski, Tobias Winkler, Carsten Perka, Michael Müller

**Affiliations:** Center for Musculoskeletal Surgery, Department of Orthopaedic Surgery, Charité-University Medicine, Berlin, Germany

## Abstract

**Aim of This Study:**

Periprosthetic joint infections (PJIs) require a special antimicrobial regimen, fundamentally different from an aseptic treatment, making a correct preoperative diagnosis essential. However, a successful preoperative microbe detection is not always possible. We wanted to find out** (1)** if a preoperative microbe detection is a prerequisite before starting a septic revision in suspected PJIs or if the preoperative diagnosis can solely be based on (para)clinical signs (persistent CRP >1 mg/dl, early X-ray loosening signs in the first 5 years, leucocytes joint aspiration >1700/*µ*l, conspicuous history, and clinical signs like redness, pain, hyperthermia, swelling, and loss of function);** (2)** if patients with and without preoperative microbe detection have a different outcome; and** (3)** if the microbial growth is the most important criterion of a multifactorial PJI definition.

**Methods:**

We included all first-line two-stage hip (49) and knee (47) revisions, performed in our department from 06/2013 on, with an available 2-year follow-up. A PJI was defined as one of the following four criteria: fistula or purulence, Krenn Morawietz type 2 or 3, joint aspirate > 2000/*μ*l leukocytes or >70% granulocytes, and microbial growth. This multifactorial PJI definition was based on the European Bone and Joint Infection Society (EBJIS). The standardized diagnostic algorithm is described in detail.

**Results:**

** (1) **24 hip and 16 knee cases were treated without preoperative microbe detection solely on the basis of a (para)clinical diagnosis (see above). In the hip 91.6% (22 of 24 cases) showed an intraoperative microbe detection. In the knee, in 68.7% (11 of 16 cases) a microbe was detected intraoperatively and in 93.7% (15 of 16) at least one secure PJI criterion could be confirmed intraoperatively.** (2) **No statistical significant (p .517) difference between patients with (n = 56, reinfection rate 8.9%) and without (n = 40, 15%) preoperative microbe detection was found in a 2-year follow-up.** (3) **Microbial growth remains the overall (pre- and intraoperatively) most important criterion (hip 95.9%; knee 89.3%), followed by Krenn Morawietz for the intraoperative diagnosis (hip 67.3%, knee 48.9%), and joint aspiration for the knee and fistula for the hip, respectively, as preoperative criteria.

**Conclusion:**

High rates of intraoperatively fulfilled EBJIS PJI criteria show that a preoperative microbe detection is not necessary before intervening in suspected PJIs. The indication for a septic revision can solely be based on (para)clinical signs. The new established diagnostic algorithm based on a multifactorial PJI definition showed high precision in finding PJIs.

## 1. Background and Aim of This Study

Periprosthetic joint infections (PJIs) are one of the most challenging complications in total joint arthroplasty. Their treatment requires a specific therapy protocol, essential for a successful infection free outcome and fundamentally different from an aseptic procedure [1]. This makes an accurate preoperative diagnosis essential. Frequently, a preoperative evidence of microbiological growth is required for the indication of infection therapy, arguing that germ detection is the most secure differentiation between an aseptic and septic condition (e.g., loosening). On the one hand overlooking of low-grade infections and false negative diagnosis as aseptic should be avoided by a preoperative microbe detection; on the other hand, an unnecessary specific PJI-treatment (including antibiotics) should be prevented in cases without an actual PJI.

Unfortunately, a preoperative microbe detection is not always reliable, useful, and/or practicable, especially in the case of low-grade infections. A microbial growth via synovial fluid out of a preoperative punctuation has a sensitivity of 45-75% and a specificity of 95% [1, 2]. This means that up to 25-55% of all existing PJIs are not found and about 5% of all detected infections can be considered false positive, making the preoperative detection not always reliable. In cases of a known preoperative antibiotic therapy, a joint aspiration is additionally not useful [3]. Finally, a preoperative microbe detection is not always practicable, because of often lacking hospital capacities, and when delaying the start of an obviously necessary revision (e.g., severe pain, acute joint dysfunction, and septic shock). The pathogenic background of the aggravated preoperative diagnosis in general is a biofilm formation, especially in chronic PJI cases, covering the prosthesis surface and protecting the bacteria from being aspirated [4].

Due to all those reasons, we believe that a preoperative microbe detection should not be a compellingly necessary precondition when intervening, with a combined antimicrobial and surgical regimen, in suspected PJIs.

1. We would like to demonstrate that the indication for septic revision can only be made on the basis of clinical and (para)clinical abnormalities, that no preoperative proof of germs is necessary, and that the final definitive diagnosis via microbe detection or another secure PJI defining criterion can be made intra/postoperatively.
2. Additionally, we would like to find out if patients with a preoperative microbe detection might have a different outcome compared to patients without a preoperatively known microbe. We therefore analyzed the reinfection rate of those two groups in a 2-year follow-up.
3. Finally, we would like to analyze which PJI definition criterion (clinical, histology, joint aspiration, and microbial growth) of a new multifactorial definition was the most often confirmed in the pre- and intraoperative diagnosis.

## 2. Methods

### 2.1. Inclusion/Exclusion Criteria

We performed this study on the example of the two-stage exchange as the most prevalent treatment type used in (suspected) PJIs at our department. Except two operations with a short interval (2 weeks), all included cases had a long interval (6-8 weeks) between prosthesis explanation and reimplantation. In the knee, a temporary arthrodesis, in the hip a girdlestone situation without a spacer was preferred after explanation.

We included all patients: (1) with a suspected PJI, (2) of a total hip/knee arthroplasty (THA, TKA) and/or hip hemiarthroplasty, (3) which received a two-stage exchange as a first-line treatment in our department, and (4) from 06/2013 on with an available 2-year follow-up.

We excluded all patients not treated completely in our hospital (e.g., prosthesis explanation in a different hospital).

### 2.2. Analysis

The data were collected retrospectively using our electronic data system (SAP). The statistical evaluation was performed using SPSS. P < .05 was considered as significant and Fisher exact test was used to determine significant differences between groups.

Of the included two-stage revisions, we worked out all cases without a preoperative microbe detection and a treatment indication based only on (para)clinical signs, of which at least 2 had to be positive [5]: (1) persistent CRP-value >1 mg/dl, (2) loosening signs in the X-ray (early loosening in the first 5 years), (3) leucocytes count >1700/*µ*l in joint aspiration, (4) conspicuous history (PJI intervention in the past), and (5) clinical signs of an infection (redness, pain, hyperthermia, swelling, and loss of function). Afterwards, we analyzed how much percent of these patients clearly fulfilled the definition of a PJI (PJI definition see below) postoperatively (only referred to the explanation procedure, first stage) by a successfully detected microbe in an intraoperative sample and/or by another PJI defining criterion. Cases with microbes detected preoperatively by aspiration or biopsies in other hospitals were considered as known preoperative microbes as well.

We compared the reinfection rate of cases with a preoperatively detected microbe with the ones without a preoperatively detected microbe. The analyzed follow-up time was 2 years, beginning after the prosthesis reimplantation. Treatment failure/reinfection was defined as a negative Delphi-based definition of success [6]:

- No wound healing with fistula, drainage, or pain, with infection recurrence caused by the same organism strain
- A subsequent surgical revision for infection after reimplantation. We extended the negative Delphi-based definition of success by the following reinfection signs: any PJI definition criterion (see below), persistent CRP-value >1 mg/dl, X-ray loosening signs, leucocytes count >1700/*µ*l in joint aspiration, and clinical signs of infection like redness, pain, hyperthermia, swelling, and loss of function
- PJI-related death (sepsis, necrotizing fasciitis)

Finally, we analyzed which PJI criterion of a multifactorial definition was the most often confirmed in the pre- and intraoperative diagnosis. Our PJI definition is based on the European Bone and Joint Infection Society (EBJIS) [7] and is similar to the Infectious Diseases Society of America (IDSA) [8]. The EBJIS Guidelines have been used increasingly in recent years in several outcome studies [9, 10]. They turned out to show a higher sensitivity for low-grade PJIs compared to the Musculoskeletal Infection Society (MSIS) [11, 12]. Following the EBJIS definition, a PJI is diagnosed if at least one of the following criteria is fulfilled:

- Clinical: sinus tract (fistula) $\underline{or}$ purulence around prosthesis
- Cell count in joint aspiration: > 2000/*μ*l leukocytes $\underline{or}$ > 70% polymorphonuclear granulocytes (PMN)
- Histology: inflammation in periprosthetic tissue (type 2 or 3 after Krenn Morawietz) [13]
- Microbial growth in synovial fluid $\underline{or}$ ≥ 2 tissue samples (in cases of high virulent microbes like Staph. aureus one sample is considered sufficient) $\underline{or}$ sonication fluid ≥ 50 CFU/ml

The results of the EBJIS were also briefly compared with the ones of the IDSA criteria. In contrast to the EBJIS, the IDSA only uses clinical features like sinus tract or purulence, histopathologic examination of the periprosthetic tissue, and positive microbe detection, but not the cell count in joint aspiration as definitive PJI criteria [8].

### 2.3. Diagnostic Algorithm

**Figure 1:**
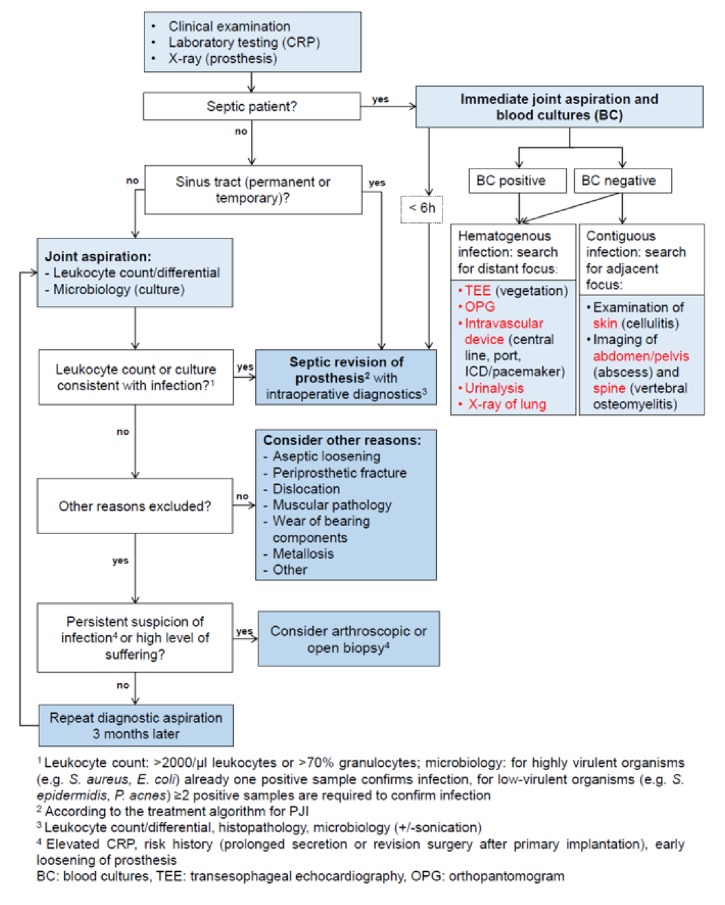
Diagnostic algorithm [2].

The idea of a multifactorial PJI definition is also present in the diagnosis algorithm used in our department (Figure 1). This algorithm was the basis of our preoperative diagnosis and following the idea that a preoperative microbe detection is not necessary when starting a septic revision in suspected PJIs. For example, the indication for a septic revision could be justified solely by finding a sinus tract or a suspicious leukocyte count in the joint aspiration. However, the indication for a septic revision also can be based on findings in septic patients via a preoperative microbe detection. Additionally, the algorithm puts a special emphasis on identifying the infectious focus.

## 3. Results

Overall, we were able to include 49 hips and 47 knee two-stage exchanges.  Table 1 is showing the rates of pre- and intraoperatively detected microbes. In the hip, we were not able to identify microbes in 48.9% (24 cases) of the cases before the prosthesis explanation took place. Of these 24 cases, 91.6% (22 of 24 cases) showed an intraoperative microbe detection, 50% (12 of 24) an intraoperatively detected microbe, and additionally an infectious periprosthetic membrane (type 2 or 3 based on Krenn Morawietz classification), and in 95.8% (23 of 24 cases) at least one PJI criterion was fulfilled intraoperatively. In the knee, in 34.0% (16 of 47 cases) a microbe could not be determined preoperatively. Of these 16 cases, a microbe could be found intraoperatively in 68.7% (11 of 16 cases), in 31.2% (5 of 16) an intraoperatively detected microbe, and additionally an infectious Krenn Morawietz membrane type were identified, and in 93.7% (15 of 16 cases) at least one PJI criterion could be confirmed intraoperatively. None of the differences is significant when comparing hip and knee.

Cases with a preoperatively detected microbe are showing a lower reinfection rate after the 2-year follow-up (8.9%, 5 of 56 cases) than cases without a known preoperative microbe (15%, 6 of 40). However, this difference is not significantly higher (p .517).

Table 2 is showing the preoperatively fulfilled (para)clinical signs in suspected hip and knee PJIs. In both hip and knee, clinical signs of an infection existed in all cases. The second most important preoperatively criterion was a persistent CRP-value >1 mg/dl in 71.4% of the hip- and 63.8% of the knee infections (p .514). A prosthesis loosening in the X-ray, which is significantly (p .013) more often present in hip (53%) than in suspected knee infections (27.6%), and a conspicuous history (45% hip, 59% knee) were also relevant parameters. Leucocytes count >1700/*µ*l in joint aspiration is the least important preoperative paraclinical sign and not showing significant differences (p .631) between hip (20.4%) and knee (25.5%).

Additionally, we analyzed the rates of pre- and intraoperatively fulfilled PJI criteria, shown in Table 3. All two-stage exchanges in the hip and 97.8% of the knee fulfilled at least one PJI criterion. Thereby, overall more PJI criteria could be confirmed intraoperatively than preoperatively in both hip (97.9%, 69.3%, p < .001) and knee (85.1%, 68.0%, p .087). The microbial growth was the overall most often fulfilled PJI criterion. In the hip 95.9% (47 of 49) and in the knee 89.3% (42 of 47) fulfilled this PJI definition either pre- or intraoperatively. Microbial detection was also the most confirmed isolated preoperative (hip 51%, knee 65.9%) and intraoperative (hip 85.7%, knee 65.9%) diagnosis criterion. The second most overall and second most intraoperatively fulfilled PJI criterion was an infectious periprosthetic membrane (Krenn Morawietz type 2 or 3). This histological PJI criterion usually only can be fulfilled intraoperatively (67.3% in the hip; 48.9% in the knee). In some cases, a preoperative diagnosis via arthroscopy is possible, too. However, this was not the case in this patient group. The third overall most fulfilled criterion (knee 44.6%; hip 30.6%) was a suspicious joint aspiration (cell count >2000/*μ*l leukocytes or > 70% PMN). Thereby, the isolated pre- (20.4%) and intraoperative (16.3%) hip rates, as well as the isolated pre- (23.4%) and intraoperative (27.6%) knee rates, were comparable. The overall least fulfilled criterion was a sinus tract (fistula) or purulence around prosthesis (hip: 22.4%; knee 8.5%). In the hip, all preoperative known fistula could be confirmed intraoperatively (22.4%), but no other additional case with purulence around the prosthesis was identified. In the knee, one additional case fulfilling this criterion could be found intraoperatively (8.5%) compared to the preoperative situation (6.3%).

Compared with the EBJIS definition results, the IDSA criteria only considered 93 of the 96 cases as a PJI. The two further cases defined via the EBJIS criteria were solely defined via the joint aspiration as an additional criterion compared to the IDSA results. One case affected the hip, the other one the knee. Both cases had no reinfection in the 2-year follow-up and no intervention because of a PJI in the past. However, in both cases infection symptoms, in one additional loosening signs in the X-ray and in the other one a suspicious joint aspiration, were found in the preoperative situation, making the decision for intervention justified. The only case neither fulfilling one EBJIS nor one IDSA criterion was a knee joint. The patient had a septic revision in the past, showed loosening signs in the preoperative X-ray, had clinical symptoms of an infection, and had an elevated CRP >1 mg/dl. The pathologist report, after the prosthesis explanation, was neither able to identify a clear infection via Krenn Morawietz (type I), nor able to rule out an infection totally because of one nonperiprosthetic tissue sample with possible signs of a low-grade infection. The patient did not have another septic revision in the 2-year follow-up.

## 4. Discussion

Septic revisions without a preoperatively detected microbe are of high clinical importance. We were able to show that over a 2.5-year-long period of time, including almost 100 two-stage exchanges, almost every second hip (48.9%), and more than every third (34.0%) knee revision, was indicated by highly clinical and paraclinical suspicion of PJI but performed without a preoperatively detected microbe. Of these operations, the rate of intraoperatively first-time detected microbes is higher in hips (91.6%) than in knee joints (68.7%). This shows that, in contrast to the hip, the intraoperative microbe detection as only PJI criterion, without preoperatively known microbe, is not sufficient for the final PJI diagnosis in knee patients. A combined diagnosis via intraoperative microbe detection (sensitivity 45-90%; specificity 92-95%) and an additionally found infectious periprosthetic membrane (Krenn Morawietz 2 or 3; sensitivity 73%; specificity 95%) is able to increase the already high sensitivity and specificity of both isolated criteria even further [1, 2]. When using this strict combined intraoperative PJI criterion, the rates in neither hip (50%) nor knee (31.2%) would be sufficient, for a septic revision in suspected PJIs, without a preoperatively known microbe. However, we consider the high rates (95.8% hip; 93.7% knee) of at least one intraoperatively found PJI criterion, in cases without preoperatively known microbe, as a sufficient proof, that the primary indication for a revision solely can be based on (para)clinical signs and that a preoperative microbe detection is no necessary prerequisite before intervening in suspected PJIs with a septic revision. The four used PJI definition criteria are showing a specify of 92-100% [1, 2] and are widely accepted as defining criteria in the EBJIS and three of them in the IDSA definition [7, 8].

The reinfection rates of cases with a preoperatively detected microbe and the ones without preoperative detection are not showing significant differences in a 2-year follow-up. However, the group with preoperative microbe detection still shows a 6.1% lower reinfection rate. Even this slightly higher rate has enormous cost for the health care system and significant clinical importance for the individual patient making an efficient and correct preoperative diagnosis a necessity [1, 14, 15]. Overall, the study shows the high importance of an efficient preoperative microbe detection. Maybe an earlier targeted and more specific antibiotic therapy might explain the different outcome. Here, further research seems promising.

Suspicious clinical presentation and a CRP-value >1 mg/dl are the most important (para)clinical PJI signs without a definitive confirmation of the diagnosis. The microbial growth remains the overall (pre- and intraoperative) most import definitive PJI criterion in hip and knee patients, followed by Krenn Morawietz for the intraoperative diagnosis, and joint aspiration for the knee, fistula for the hip respectively, as preoperative diagnosis criterion. However, microbial growth as only definitive criterion is not sufficient. It failed to include 4.1% of all hip infections and even 8.5% of all knee PJIs, defined by another criterion. This shows the necessity of a multifactorial PJI definition and diagnosis, which has also become the standard in modern guidelines like MSIS, IDSA, and EBJIS.

For a further comparison and interpretation of the different rates of fulfilled PJI criteria in hips and knees, detailed diagnosis information would be necessary, especially the absolute number of performed joint aspirations, arthroscopies, gathered tissue samples, and histology membrane samples (Krenn Morawietz). Without this information, a final comparison between single hip and knee results is not possible or useful. For example, a specific PJI criterion rate can be higher in one joint type because of a more intense diagnosis (e.g., more preoperative arthroscopies in knee PJIs could lead to a higher preoperative rate of the microbial growth criterion), while in other criteria structural differences might explain the results. However, such a detailed comparison was not the aim of this study. We wanted to analyze which PJI criterion was the most important in a daily clinical routine, under consideration of different diagnostic intensity. As only criterion, further diagnosis information is not necessary, when evaluating the clinical definition (fistula, purulence). This criterion is primarily a first view diagnosis and thus not showing major differences in the level of diagnosis. Here, higher rates could be found in hip PJIs (p .091). This could be explained by the fact that the hip is a larger joint, making a clinical view diagnosis (fistula, purulence) easier.

In the last years, a general preference towards algorithm systems for the diagnosis of PJIs could be seen. The developed algorithms vary, depending on their setting and aim. Some algorithms focus on hospitals without specialization on PJIs and present systems with as few steps as possible [17], while others put a stronger focus on maximal precision in a scientific analysis and research context [18]. Our diagnostic algorithm is showing high accuracy in finding PJIs. All two-stage exchange operations in the hip and 97.8% of the knee fulfilled at least one PJI criterion.

## 5. Conclusion

A preoperative microbe detection is not necessary before intervening in suspected PJIs. The indication for a septic revision can solely be based on clinical and paraclinical signs (persistent CRP-value > 1 mg/dl, conspicuous history, loosening signs in the X-ray, early loosening in the first 5 years, leucocytes count >1700/*µ*l in joint aspiration, and clinical signs). Thereby, suspicious clinical presentation and a CRP-value >1 mg/dl are still the most important (para)clinical signs. Cases with a preoperatively detected microbe are showing slightly better results compared to cases without a known preoperative microbe. The pre- and intraoperative microbe detection remains the most import PJI definition and diagnosis criterion. Our new established diagnostic algorithm is showing high accuracy in finding PJIs. However, a detailed analysis of the algorithm will be necessary for a final evaluation. Overall, the results found in this study might be helpful when making future decisions in unclear preoperative situations.

## Figures and Tables

**Table 1:** Rates of pre- and intraoperatively detected microbes.

Number of two-Stage exchanges	Hip	Knee	P
Total Number of two-Stage exchanges	49	47	-

Two-stage exchanges without a preoperatively detected microbe	24 (48.9%)	16 (34.0%)	.153

Two-stage exchanges without a preoperatively detected microbe, but an intraoperatively detected microbe	22 of 24 (91.6%)	11 of 16 (68.7%)	.094

Two-stage exchanges without a preoperatively detected microbe, but intraoperatively detected microbe and additionally found Krenn Morawietz type 2 or 3	12 of 24 (50%)	5 of 16 (31.2%)	.332

Two-stage exchanges without preoperatively detected microbe, but at least one intraoperatively fulfilled PJI criterion	**23 of 24 (95.8%)**	**15 of 16 (93.7%) **	** .999**

**Table 2:** Preoperatively fulfilled (para)clinical signs in suspected hip and knee PJIs.

Preoperative (para-)clinical signs	Hip (n = 49)	Knee (n = 47)	P
Persistent CRP-value >1 mg/dl	35 of 49 (71.4%)	30 of 47 (63.8%)	.514

Loosening signs in the X-Ray, *especially early* loosening in the first 5 years (ranging from decent loosening to entire migration)	26 of 49 (53.0%)	13 of 47 (27.6%)	.013

Leucocytes count >1700/*µ*l in joint aspiration (Not determinable for every patient)	10 of 49 (20.4%)*∗*	12 of 47 (25.5%)*∗*	.631

Conspicuous history (PJI intervention in the past)	22 of 49 (45%)	28 of 47 (59%)	.160

≥ 1 clinical signs of an infection (redness, pain, hyperthermia, swelling, loss of function)	49 of 49 (100%)	47 of 47 (100%)	-

*∗* not available for each patient.

**Table 3:** Rates of pre- and intraoperatively fulfilled PJI criteria, following the 4 PJI definitions of the EBJIS.

Hip, n=49	Overall (pre – or Intraoperative)	preoperative	intraoperative (explanation)
Sinus tract (fistula) or purulence	11 of 49 (22.4%)	11 of 49 (22.4%)	11 of 49 (22.4%)

>2000/*μ*l leukocytes or >70% PMN	15 of 49 (30.6%)*∗*	10 of 49 (20.4%)	8 of 49 (16.3%)*∗*

Krenn Morawietz type 2 or 3	33 of 49 (67.3%)*∗*	0	33 of 49 (67.3%)*∗*

Microbial growth	**47of 49 (95.9%)**	**25 of 49 (51%)**	**43 of 49 (85.7%) **

At least one PJI criterion	49 of 49 (100%)	34 of 49 (69.3%)	48 of 49 (97.9%)

**Knee, n=47**	**Overall (pre – or ** **Intraoperative) **	**preoperative**	**intraoperative (explanation) **

Sinus tract (fistula) or purulence	4 of 47 (8.5%)	3 of 47 (6.3%)	4 of 47 (8.5%)

>2000/*μ*l leukocytes or >70% PMN	21 of 47 (44.6%)*∗*	11 of 47 (23.4%)*∗*	13 of 47 (27.6%)*∗*

Krenn Morawietz type 2 or 3	23 of 47 (48.9%)*∗*	0	23 of 47 (48.9%)*∗*

Microbial growth	**42 of 47 (89.3%)**	**31 of 47 (65.9%)**	**31 of 47 (65.9%)**

At least one PJI criterion	46 of 47 (97.8%)	32 of 47 (68.0%)	40 of 47 (85.1%)

*∗* not available for each patient

## Data Availability

The data used to support the findings of this study are available from the corresponding author upon request.
